# One-Pot Synthesis of Mechanically Robust Eutectogels via Carboxyl-Al(III) Coordination for Reliable Flexible Strain Sensor

**DOI:** 10.3390/gels11120963

**Published:** 2025-11-28

**Authors:** Zhenkai Huang, Yutao Song, Shanzheng Zhao, Peijiang Liu, Jianping Peng

**Affiliations:** 1 School of Materials and Energy, Foshan University, Foshan 528000, China; hzk@fosu.edu.cn; 2 School of Environment and Chemical Engineering, Foshan University, Foshan 528000, China; 15535522031@163.com (Y.S.); m19926248648@163.com (S.Z.); 3 Science and Technology on Reliability Physics and Application Technology of Electronic Component Laboratory, The Fifth Electronics Research Institute of the Ministry of Industry and Information Technology, Guangzhou 510610, China

**Keywords:** deep eutectic solvent, eutectogel, flexible sensing, mechanical strength, ionic conductivity

## Abstract

This work presents a facile one-pot synthesis strategy for highly strong and tough eutectogels. The exceptional mechanical performance (average stress: 3.8 MPa; average strain: 920%) stems from synergistic trivalent aluminum(III)–ligand coordination crosslinking and extensive hydrogen bonding within the network. The optimized proportion has achieved a balance between mechanical stress and strain. Reversible bonding enables rapid energy dissipation and elastic recovery. A flexible strain sensor fabricated from this eutectogel demonstrates high sensitivity (gauge factor > 2) and ultrafast response (200 ms), validating its potential as low-cost electronic skin. This research provides a foundational framework for developing sustainable, high-performance flexible.

## 1. Introduction

Hydrogel-based flexible sensors have emerged as pivotal components in soft robotics, wearable electronics, and artificial intelligence systems due to their intrinsic biocompatibility and tunable mechanical properties [1,2,3,4,5]. Conventional hydrogels, however, suffer from critical limitations, including dehydration-induced performance degradation, compromised flexibility at subzero temperatures, and electrode corrosion during prolonged operation [6,7,8]. While ionogels partially mitigate these issues, their high production costs and inherent toxicity impede scalable implementation [9,10,11,12,13,14,15]. Consequently, there remains an urgent need to develop next-generation gel materials that simultaneously satisfy requirements for environmental sustainability, cost-effectiveness, and robust electromechanical performance [16,17].

Deep eutectic solvents (DES) have recently gained attention as green alternatives to address these challenges [18,19]. Composed of a hydrogen bond acceptor (HBA, e.g., choline chloride, ZnCl_2_) and a hydrogen bond donor (HBD, e.g., ethylene glycol, propylene glycol, phytic acid, and urea), DES represent a novel class of environmentally benign liquid media [20,21,22,23]. These solvents exhibit properties analogous to ionic liquids, including low volatility, minimal toxicity, biodegradability, excellent thermal stability, ionic conductivity, and a broad electrochemical window [24]. Moreover, DES offer distinct advantages over ionic liquids, such as enhanced biocompatibility, lower cost, and simplified preparation [18,19]. Consequently, DES are regarded as promising alternatives to conventional ionic liquids.

Immobilizing DES within polymer networks yields deep eutectic solvent gels (eutectogels), a rapidly emerging class of soft ionic conductors [25,26,27]. These materials emerge as green, low-cost, and biocompatible alternatives to hydrogels and ionogels. Leveraging the elasticity of polymeric matrices and the inherent favorable properties of DES, eutectogels demonstrate significant potential for flexible electronics and smart sensing applications [28,29,30,31,32]. Nevertheless, current eutectogel development faces challenges, notably relatively low mechanical strength (typically below 1 MPa) and the fabrication complexity often required for mechanical enhancement. Eutectogels fabricated using common polymer networks—including poly(acrylic acid) (PAA), polyacrylamide (PAM), poly(vinyl imidazole) (PVI), and poly(vinyl alcohol)/poly(N-(2-hydroxyethyl)acrylamide) (PVA/PHEAA)—consistently exhibit mechanical strengths within the sub-1-MPa range [20,33,34,35]. For instance, PAA-based eutectogels achieve mechanical strengths between 0.2 and 0.6 MPa [36,37], while PVI-based systems report values near 0.5 MPa [38]. Although sufficient for certain applications, these mechanical properties remain inadequate for demanding scenarios involving harsh external stresses.

Several studies on high-performance eutectogels have been reported. For instance, Zhang et al. constructed a mechanically robust deep eutectic organogel by dispersing a PVA polymer network into a deep eutectic solvent, leveraging the partial crystallization of PVA [39]. However, the preparation processes for these mechanically robust eutectogels are often complex, requiring solvent exchange or multiple polymerizations to build double-network structures.

Recently, the successful application of metal-coordination interactions in the field of hydrogels has provided a feasible strategy for further enhancing the mechanical properties of eutectogels. This approach involves introducing robust metal-coordination interactions into hydrogel systems, leading to the preparation of a series of mechanically tough metal-coordination crosslinked hydrogels. For example, Zhao et al. prepared a mechanically robust hydrogel via Fe^3+^ coordination with acrylic acid [40]. Moreover, Yu et al. designed and prepared a series of hydrogels formed through coordination interactions using different ions with acrylic acid functional groups, investigating the effects of different metal ions on the gel’s mechanical properties [41]. Inspired by this strategy, the introduction of metal-coordination interactions into eutectogels is expected to enable a straightforward and efficient preparation method, significantly enhancing their mechanical properties while preserving their excellent conductivity and other advantageous characteristics.

Herein, we report a facile one-pot synthesis strategy for producing highly strong and tough eutectogels. The exceptional mechanical robustness originates from synergistic interactions of two key mechanisms within the polymer network: robust trivalent aluminum ion (Al^3+^)–ligand coordination crosslinking and extensive intermolecular hydrogen bonding. By optimizing the mass ratios of acrylic acid (AA) to DES (10:1) and anhydrous aluminum chloride to AA (0.12:1), we synthesized a eutectogel exhibiting an exceptional stress–strain balance (average stress: 3.8 MPa; average strain: 920%)—performance exceeding conventional hydrogel benchmarks. This design leverages reversible hydrogen bonding and ion–dipole interactions to enable rapid energy dissipation and elastic recovery. A flexible strain sensor based on this eutectogel demonstrates high sensitivity (gauge factor >2) and ultrafast response (200 ms), validating its potential as low-cost electronic skin. This research establishes a foundational framework for developing sustainable, high-performance flexible sensors that overcome persistent limitations in existing gel technologies.

## 2. Results and Discussion

### 2.1. Design and Preparation of Eutectogel

**Figure 1:**
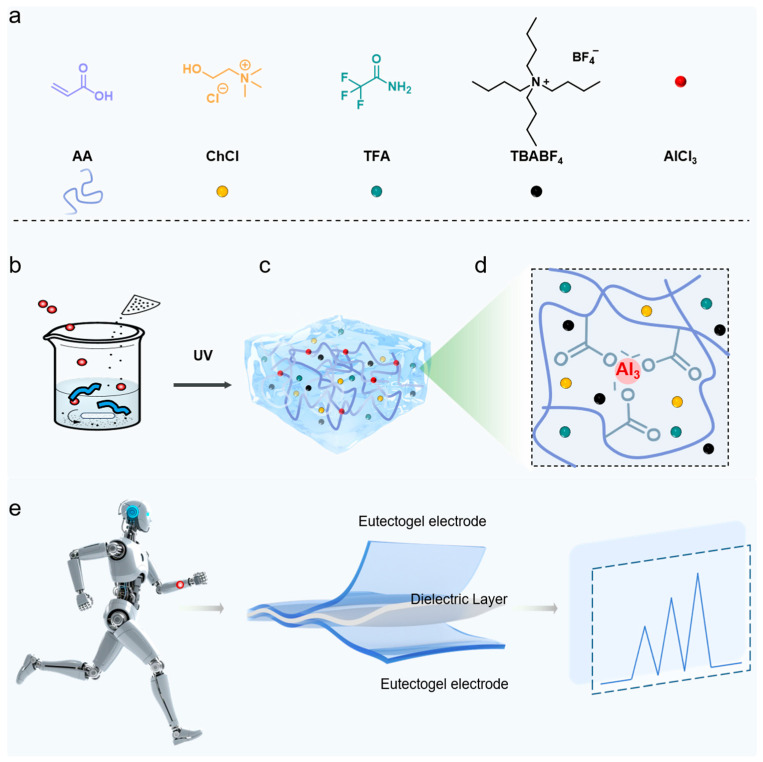
Illustration of the preparation process and network structure of the tough eutectogel crosslinked by carboxyl–Al^3+^ coordination. (**a**) Raw materials for the eutectogel. (**b**) Preparation procedures of the eutectogel. (**c**) Polymer network structure of the eutectogel and (**d**) magnified view of the carboxyl–Al^3+^ coordination. (**e**) Schematic illustration of the eutectogel functioning as a flexible strain sensor for monitoring joint movement signals.

The design and preparation of the eutectogel are illustrated in Figure 1a–d. Eutectogels are formed via the in situ polymerization of polymerizable monomers within a deep eutectic solvent (DES). Prior to polymerization, the polymerizable monomer acrylic acid (AA) and Al^3+^ metal ions are dissolved in the DES. In the presence of a UV-initiator, photoinitiated in situ polymerization converts the monomers into a polymer network, which is physically crosslinked into a robust network via metal-coordination interactions with Al^3+^ ions. These physical crosslinks enable the polymer network to integrate into a cohesive structure, dissipating a substantial amount of mechanical energy under external load, thereby enhancing the mechanical properties of the gel. Furthermore, the DES component within the eutectogel acts as a dispersion medium and can undergo directional movement within the polymer network under an applied electric field, thereby conferring conductivity to the eutectogel. Consequently, such a eutectogel, possessing both favorable mechanical properties and conductivity, represents an ideal material for constructing flexible strain sensors (i.e., electronic skin), enabling real-time signal monitoring of motion information from human or robotic joints (Figure 1e).

**Figure 2:**
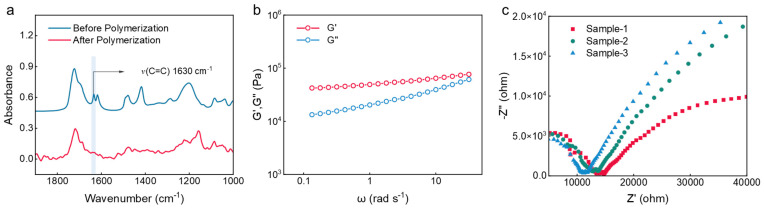
FTIR, rheology, and electrochemical impedance spectroscopy of eutectogel. (**a**) FTIR spectra of the eutectogel before and after polymerization. (**b**) Storage modulus (G′) and loss modulus (G″) of the polymerized eutectogel as a function of frequency. (**c**) Electrochemical impedance spectroscopy (EIS) plots of three eutectogel samples from different batches with identical composition.

To confirm the formation of the polymer network within the eutectogel, we characterized the polymerization process using Fourier Transform Infrared (FTIR) spectroscopy, as shown in Figure 2a. The FTIR spectrum of the precursor solution exhibited a distinct characteristic peak near 1630 cm^−1^, attributed to the C=C bonds. This peak markedly disappeared in the spectrum of the post-polymerization eutectogel. This stark contrast confirms the successful conversion of most monomers in the precursor solution into the polymer network via the polymerization reaction. To further verify the metal-coordination interactions between Al^3+^ ions and the polymer network, rheological tests were performed, as presented in Figure 2b. The rheological results demonstrate that the storage modulus (G′) of the eutectogel exceeds its loss modulus (G″) across the entire tested frequency range, indicating that the eutectogel is an elastic solid, which suggests the formation of a firmly crosslinked network internally. Moreover, the relatively high values of both G′ and G″ also imply the presence of strong interactions within the eutectogel, granting it a potent ability to dissipate external mechanical energy. Figure 2c shows the electrochemical impedance spectroscopy (EIS) plots of different batches of eutectogels prepared with the same formulation, used to measure their ionic conductivity. The experimental results indicate that the abundant DES within the eutectogel possesses good fluidity, thus endowing the eutectogel with favorable ionic conductivity. The ionic conductivity, calculated from the intrinsic resistance, is approximately 1.3 × 10^−2^ mS cm^−1^, and different batches exhibit similar intrinsic resistance. Combining the ionic conductivity and excellent elasticity, the eutectogel demonstrates broad potential application value in the field of flexible sensor devices.

### 2.2. Mechanical Performance of Eutectogel

**Figure 3:**
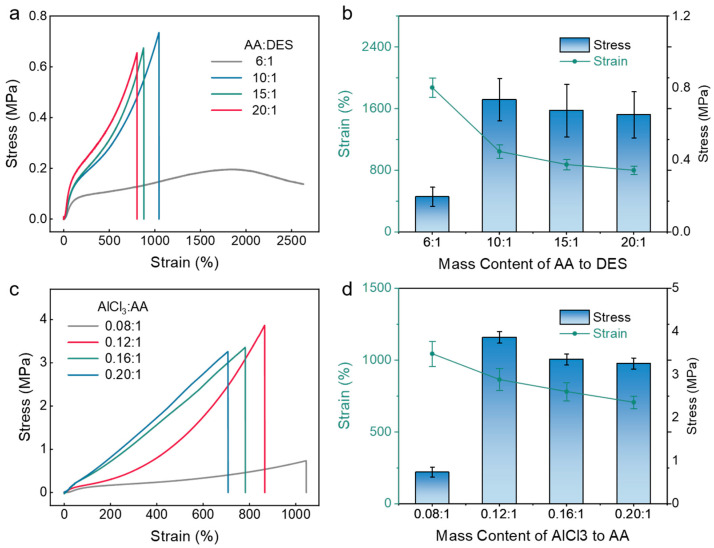
Mechanical performance of eutectogels with different anhydrous aluminum chloride-to-AA molar ratios. (**a**) Strain–stress curves of eutectogels with different AA-to-DES molar ratios from 6:1 to 20:1. (**b**) Summary of fracture strength and elongation at break for eutectogels with different AA/DES ratios. (**c**) Strain–stress curves of eutectogels with different AlCl_3_-to-AA molar ratios from 0.08:1 to 0.20:1. (**d**) Summary of fracture strength and elongation at break for eutectogels with different Al^3+^/AA ratios.

The eutectogels have tunable mechanical properties. Analysis from Figure 3a above shows that the eutectogel with a mass ratio of 6:1 for AA and DES has an average stress of 0.22 MPa and an average strain of 2500%. With the increase in AA content, the fracture strength of the eutectogel initially increased to a maximum value of 0.74 MPa and subsequently decreased. Furthermore, as the AA content increased, the elongation at break gradually decreased from 2500% to 820% (Figure 3b). In summary, among the four groups of eutectogels with different AA and DES ratios, the eutectogel with the highest average stress of 0.74 MPa had an average strain of near 1100%. And the eutectogel with the highest average strain of 2500% had an average stress of only 0.22 MPa. The eutectogel with a mass ratio of 10:1 exhibited relatively balanced average stress and strain among the four different ratios, meeting the requirements for practical application. This is attributed to the gradual increase in polymer network concentration within the eutectogel as the AA content rises, leading to an increase in the modulus of the gel. Conversely, the higher proportion of the polymer network results in a reduction in the deformability of eutectogel. This trade-off between increasing modulus and decreasing stretchability reaches a moderate balance at the 10:1 ratio, yielding the highest mechanical strength of 0.74 MPa. Therefore, the mass ratio of 10:1 for AA and DES was selected for subsequent experiments.

After finding the optimal ratio of AA and DES solution, to enhance the average stress of the eutectogel with the 10:1 mass ratio of AA and DES, the amount of anhydrous aluminum chloride (AlCl_3_) can be introduced. The ratio of anhydrous aluminum chloride to AA was investigated to enhance the average stress of eutectogel, facilitating subsequent testing. As shown in Figure 3c, analysis shows that, as the Al^3+^-to-monomer ratio in the eutectogel increases from 0.08:1 to 0.20:1, the fracture strength initially increases and subsequently decreases. The experimental results show that, with increasing Al^3+^ ion content, the Young’s modulus of the eutectogels significantly increase, with the gradual decrease in elongation at break. This reveals the significant role of metal-coordination interactions in enhancing the mechanical properties of the eutectogel. This enhancement mechanism originates from the higher Al^3+^ concentration increasing the density of metal-coordination interactions within the polymer network. In the eutectogel, Al^3+^ ions act as dynamic cross-linkers, forming robust metal-coordination interactions with different polymer chains, thereby cross-linking them into a tough three-dimensional network structure. Moreover, due to the strong bond energy of metal-coordination bonds, they gradually unravel and break during the stretching of the eutectogel under external stress, dissipating a significant amount of mechanical energy. In summary, the eutectogel with AlCl_3_-to-AA mass ratio of 0.12:1 exhibited the best comprehensive mechanical performance, with an average stress reaching 3.8 MPa and an average strain reaching 920% (Figure 3d). Therefore, the optimal eutectogel formulation for this experiment is a mass ratio of 10:1 for AA and DES solution and a mass ratio of 0.12:1 for anhydrous aluminum chloride and AA. This proportion was used to complete the subsequent cyclic tensile mechanical performance testing.

It is noteworthy that our eutectogel demonstrates exceptional mechanical properties, making it comparable to several commercial products. Conventional commercial hydrogels typically exhibit elongation at break values ranging from 100% to 2000% and fracture strengths generally below 2 MPa, which are inferior to those of our eutectogel. Although the fracture strength of our eutectogel (~4 MPa) is lower than that of tire rubber (20 MPa), it is comparable to common silicone rubber (4–10 MPa). In terms of elongation at break, the eutectogel (~1000%) significantly surpasses tire rubber (500%) and is comparable to silicone rubber (~1000%). These comparative results further highlight the advantage of our eutectogel in terms of overall mechanical performance.

### 2.3. Cyclic Tensile Performance Testing

Cyclic tensile testing evaluates the changes in mechanical properties that materials undergo during repeated loading and unloading processes. It is primarily used to measure the resilience, energy dissipation capability, and fatigue characteristics of the eutectogel. During cyclic tensile testing, the sample is stretched to a maximum stress, then released and restored to its original state, followed by the next stretch cycle. Usually, the resilience of eutectogels is mainly judged by its hysteresis loop and hysteresis ratio [10,42]. The energy dissipation capability of eutectogels during cyclic tensile testing can indicate their internal friction and viscoelastic behavior under repeated loading and unloading. High energy dissipation capability means the material can effectively absorb energy during deformation, thereby resulting in a low mechanical strength of the next cycle [42].

**Figure 4:**
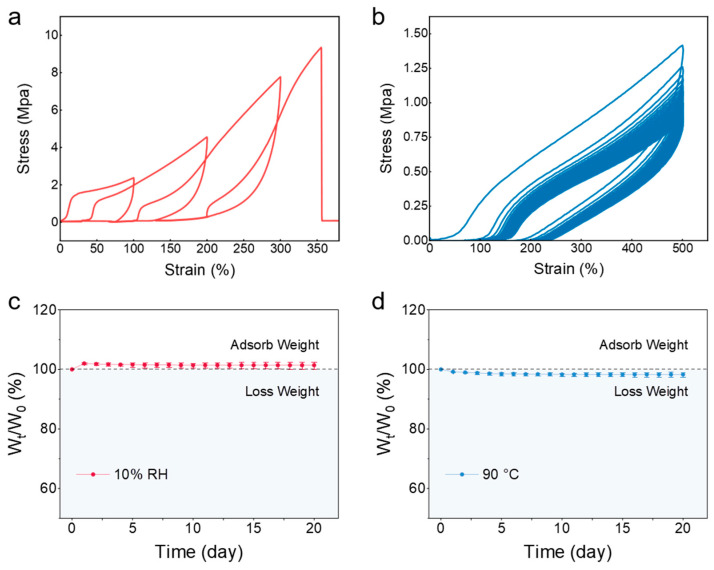
(**a**) Cyclic stress–strain curve of eutectogel under different strains. (**b**) Stress–strain curves of eutectogel with continuous 30 cycles of loading under 100% strain. (**c**) Weight change curve of the eutectogel over time under a 10% relative humidity environment. (**d**) Weight change curve of the eutectogel over time in a 90 °C environment.

As shown in Figure 4a, cyclic tensile testing was performed on the eutectogel with the optimal ratio, and its hysteresis behavior was observed and analyzed. The hysteresis effect of eutectogels can be observe during loading–unloading mechanical test. The hysteresis effect refers to the phenomenon where the stress–strain curves during loading and unloading do not coincide, forming a “hysteresis loop”. During loading, the material absorbs energy, while, during unloading, less energy is released than was absorbed, resulting in partial energy loss. Experimental results show that the eutectogel with optimized proportion exhibits good elasticity and relatively low residual strain and hysteresis ratio. After stretching the sample to 350% strain (within 1 min), it could quickly recover to its initial length. This indicates that, in the eutectogel, hydrogen bonds are typically dynamic, meaning they can form and break rapidly during loading and unloading. The good elastic properties suggest that, after stress is applied, these hydrogen bonds can quickly reconnect after breaking, restoring the material’s original shape. During stretching, metal–ligand coordination interactions and hydrogen bonds within the polymer matrix and DES temporarily break, allowing the eutectogel to dissipate part of the applied energy, reducing stress concentration and potential structural damage. This mechanism helps the eutectogel exhibit better elasticity under dynamic loading conditions. The dynamic nature of hydrogen bonds and metal–ligand coordination interactions allows the gel to adapt to different load situations without undergoing permanent deformation under small stresses. Furthermore, after molecular chains are straightened under stretch, they recover their initial conformation with good flexibility. This aligned state can promote interactions between molecular chains, helping to re-establish entanglements in the molecular network. This reconstruction helps provide additional resistance in subsequent loading processes. Nevertheless, because the reconstruction of interactions and conformational recovery in the eutectogel system require time, it leads to the cyclic stress–strain curves exhibiting typical hysteresis behavior.

As shown in Figure 4b, the optimal ratio eutectogel underwent 30 consecutive cyclic tensile tests at 100% strain. It can be observed that, as the number of cycles increases, the tensile strength of the eutectogel gradually decreases and the decline in mechanical strength gradually decreases and reaches stability. The eutectogel with optimized proportion exhibits good elasticity and relatively low residual strain. However, the presence of hysteresis loops indicates noticeable energy dissipation during cyclic loading, which is common in dynamically crosslinked networks involving reversible bonds. The decrease in tensile strength after the first cycle and stabilization in subsequent cycles can be attributed to the reason that the initial cycle leads to irreversible breakdown of some weaker bonds or network structures, after which the system reaches a new stable state. The overlapping curves in the subsequent cycles demonstrate stable and repeatable viscoelastic behavior after this initial stabilization.

### 2.4. Environmental Stability of Eutectogel

The environmental stability of the eutectogel was assessed by monitoring its mass change under different conditions. Initially, at a relatively low humidity (10% RH), the eutectogel exhibited a slight ability to absorb moisture from the air. As shown in Figure 4c, after equilibrating for three days, the mass of the eutectogel stabilized and maintained a net mass increase of approximately 2–3% throughout the subsequent testing period. This suggests that the eutectogel possesses a remarkably good capacity to retain the DES, with no observed leakage or exudation of the dispersion medium. Furthermore, under more severe conditions (heating at 90 °C), the mass of the eutectogel also remained stable, showing a gradual decrease of only about 2% over the first 7 days, and then remained constant for the following 13 days (Figure 4d). These stability tests under different environments demonstrate the superior environmental stability of the eutectogel compared to conventional hydrogels.

### 2.5. Sensing Performance Testing

#### 2.5.1. Mechanical–Electric Sensing Performance of the Flexible Strain Sensor

This section designed an ionotronic resistive flexible pressure/strain sensor device. Such devices that mimic human touch are also called electronic skin, and devices that use mobile ions for charge transmission or electrical signal acquisition are also called ionotronic skin. Bionic flexible pressure sensors undergo changes in resistance value with the degree of deformation when subjected to external forces. The assembled sensor was placed on an electronic universal material testing machine, and a preset strain level was applied. By connecting its upper and lower electrodes to an LCR digital bridge, changes in resistance (R) can be recorded in real time. These changes can be used to calculate the real-time strain (S) or pressure (P) experienced by the sensor. The relative resistance change value $\left(\frac{∆R}{R_{0}}\right)$ is calculated as follows [43,44]:

(1) $$\frac{∆R}{R_{0}}=\left(\frac{R}{R_{0}}-1\right)\times 100\%$$

The gauge factor is defined as:

(2) $${GF=\frac{∆R}{PR}}_{0}$$

where R: real-time resistance value;

R_0_: initial resistance value;

ΔR: relative resistance difference;

GF: gauge factor of the sensor;

P: pressure value.

**Figure 5:**
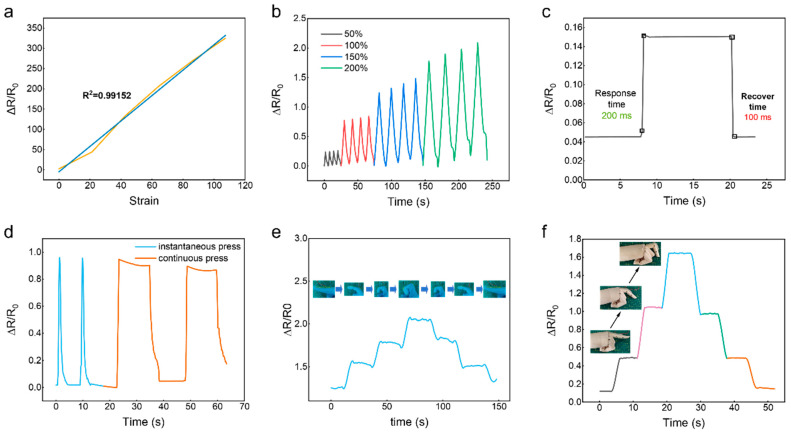
(**a**) Relative resistance changes of the flexible sensor under gradually increasing strain, the orange curve represents the experimental data, while the blue curve shows the fitted results. (**b**) Relative resistance changes of the flexible sensor under different cyclic strain. (**c**) Response time of the eutectogel-based flexible strain sensor. (**d**) Effects of applied pressure at different levels and rates on the sensor. (**e**) Relative resistance changes of the eutectogel strain sensor during finger bending. (**f**) Relative resistance changes of the eutectogel strain sensor during robotic finger bending.

As shown in Figure 5a, the relative real-time resistance change (ΔR/R_0_) of the sensor was monitored under strains ranging from 0 to 100%. The eutectogel-based flexible strain sensor showed a linear strain–resistance relationship (y = 3.08x + 0.02), simplifying signal calibration. The gauge factor within strain ranges of 0–100% was calculated as 3.03, indicating linear sensitivity. Reversible signals were observed at low (50% and 100%) and high (150–200%) strains (Figure 5b). The response and relaxation times of the sensor were about 200 and 100 ms, respectively (Figure 5c). The flexible sensor based on eutectogel is stable, fast, and reproducible, exhibiting reliable and accurate loading–unloading cyclic electrical signal curves across different strain ranges. The gauge factor of 3.03 and response time of 200 ms demonstrated by our eutectogel-based sensor are comparable or superior to many recently reported flexible strain sensors based on eutectogels or other ionic conductors [5,9,15,38].

#### 2.5.2. Real-Time Sensing Performance of the Flexible Strain Sensor

Based on the excellent response performance of our eutectogel-based strain sensor, it is inferred that our sensor might be able to mimic some functions of human skin, enabling reliable monitoring of pressing. Flexible sensing technology holds potential for broad applications in the future, including physiological information monitoring and motion capture. In particular, low-cost wearable flexible optical/electrical sensors are expected to be widely popularized, thereby facilitating daily life [45,46]. To assess wearable sensor viability, devices were affixed to human joints for motion monitoring. Notably, our flexible strain sensor exhibits responsiveness to compressive stress. Applied pressure elevated resistance, while pressure removal returned readings to baseline (Figure 5d). The first two presses involved quickly pressing and releasing a weight on the sensor, while the last two involved pressing the weight on the sensor for a sustained period without releasing. Because the first two presses were fast, the spikes generated by the rapid bending of the sensor were recorded as “points”. For the last two, because the pressing duration was long, the broad signal peaks caused by prolonged bending of the sensor were recorded as “lines”.

Resistive signatures further distinguished varied press durations. Correspondingly, cyclic bending–straightening motions produced characteristic resistive profiles (Figure 5e). Periodically modulated resistance during robotic finger bending–straightening cycles is presented in Figure 5f. Data demonstrated that resistive variations precisely followed angular displacement as bending increased from 30° to 90°, stabilizing during static angle maintenance. Signal amplitudes correlated with deformation parameters, enabling clear differentiation of both micro- and macro-scale movements. This result illustrates that our sensor responded quickly and accurately to pressure changes.

#### 2.5.3. Data Transmission of Eutectogel-Based Flexible Strain Sensor via Morse Code

**Figure 6:**
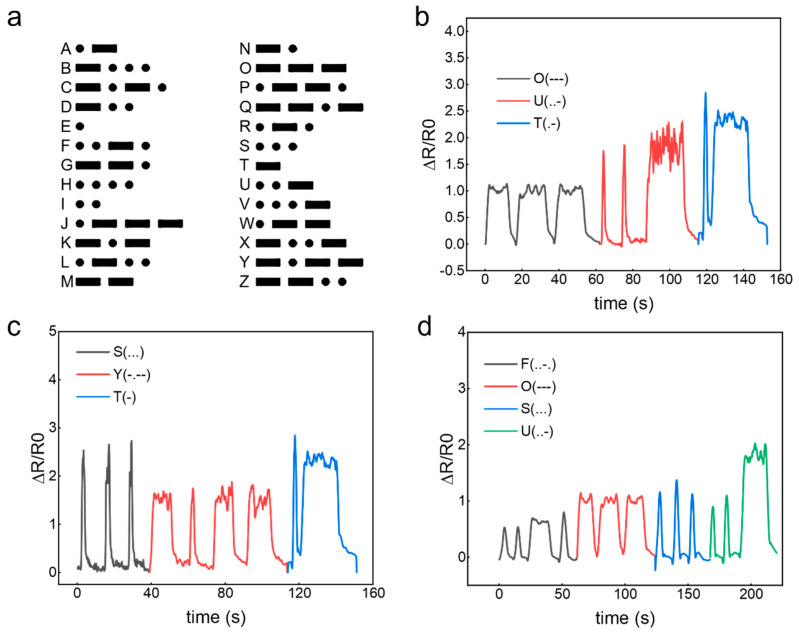
Lexical pattern recognition using Morse code: (**a**) standard alphabetic encoding, (**b**) “OUT” signal interpretation, (**c**) “SYT” signal interpretation, (**d**) “FOSU” signal interpretation.

Advanced signal processing capabilities were demonstrated through Morse code decoding. Dual-state pressure actuation (sustained vs. instantaneous) yielded distinct signal profiles corresponding to alphanumeric sequences (“OUT”, “SYT”, and “FOSU”) (Figure 6a–d). These results confirm the applicability of our eutectogel in high-fidelity wearable sensing systems when evaluated against standardized performance metrics.

## 3. Conclusions

In summary, this study presents a facile one-pot synthesis strategy for mechanically robust eutectogels through synergistic carboxyl–Al^3+^ coordination crosslinking. By optimizing the mass ratios of acrylic acid to DES (10:1) and AlCl_3_ to AA (0.12:1), we achieved a eutectogel with exceptional mechanical performance (average tensile stress: 3.8 MPa; strain: 920%)—a significant advancement beyond existing sub-1 MPa eutectogel systems. The polymer matrix combining dynamic trivalent aluminum ion–ligand coordination and extensive hydrogen bonding, enables rapid energy dissipation and elastic recovery, as evidenced by low hysteresis ratios and stable cyclic tensile behavior (30 cycles at 100% strain). The resulting eutectogel serves as an effective ionic conductor for flexible strain sensors, demonstrating high sensitivity (gauge factor > 2), ultrafast response/relaxation (200 ms/100 ms), and reliable signal stability across diverse deformations (0–200% strain). Practical applicability was validated through real-time monitoring of human joint movements and robotic finger actuation, where resistive signatures accurately tracked dynamic bending angles. Furthermore, Morse code signal transmission experiments confirmed its capability for sophisticated lexical pattern recognition. The carboxyl–Al^3+^ coordination strategy offers a scalable, fabrication-simplified pathway toward next-generation biocompatible electronics, with promising potential in wearable health monitoring, soft robotics, and human–machine interfaces. In the future, this study could be extended by integrating its UV-triggered curing strategy with UV-assisted 3D printing techniques, thereby expanding its processability and further enhancing its applicability in the field of flexible electronics.

## 4. Materials and Methods

### 4.1. Instruments

Tensile properties were measured at 25 °C and 60% relative humidity (RH) using a universal testing machine (LD22.102, 100-N load cell, Lishi Scientific Instrument Co., Ltd., Shanghai, China) at 100 mm/min. Dumbbell-shaped specimens (15 mm × 2 mm × 2 mm, gauge length: 5 mm) were tested, with Young’s modulus derived from the 0–10% strain region. Tests were repeated in at least triplicate. Cyclic tensile and compression tests were conducted at 100 mm/min and 5 mm/min, respectively. Compression tests were analyzed using cylindrical samples (8 mm × 2 mm). Optical image (CM30W-HK810, AOSVI Co., Ltd., Nanjing, China) was employed to visualize surface topography in 200 μm thick films. The optical images were captured by a digital camera (DJI Osmo Pocket Co., Ltd., Shenzhen, China). An electrochemical workstation with computer-controlled system (CHI 660E, Shanghai Lishi Scientific Instrument Co., Ltd., Shanghai, China), UV curing lamp (36 W × 2, Philips Co., Ltd., Shenzhen, China), and LCR digital bridge (TH2830, Changzhou Tonghui Electronics Co., Ltd., Changzhou, China) was used.

### 4.2. Chemicals

Acrylic acid (98%, Shanghai Adamas Reagent Co., Ltd., Shanghai, China), anhydrous aluminum chloride (99%, Shanghai Titan Technology Co., Ltd., Shanghai, China), choline chloride (99%, Shanghai Adamas Reagent Co., Ltd.), 2-hydroxy-4′-(2-hydroxyethoxy)-2-methylpropiophenone (I-2959) (99%, Shanghai Adamas Reagent Co., Ltd.), trifluoroacetamide (98%, Shanghai Adamas Reagent Co., Ltd.), and tetrabutylammonium tetrafluoroborate (98%, Shanghai Adamas Reagent Co., Ltd.) were used.

### 4.3. Synthesis of DES Solution

A predetermined quantity of trifluoroacetamide (TFA) and tetrabutylammonium tetrafluoroborate (N_4444_BF_4_), corresponding to a molar ratio of 2:1 (for instance, 1 g of TFA and 1.51 g of N_4444_BF_4_), was weighed into a vial and subsequently stirred at 50 °C with a rotation speed of 150 r/min until a clear solution formed. Separately, a predetermined quantity of acrylic acid (AA) and (ChCl) choline chloride, also at a molar ratio of 2:1 (for example, 1 g of AA and 0.97 g of ChCl), was weighed into a vial and stirred at room temperature with a rotation speed of 150 r/min until a transparent solution was obtained. A predetermined amount of aluminum chloride (AlCl_3_) was then added to this solution and stirred under a rotation speed of 150 r/min until complete dissolution occurred, producing a transparent, homogeneous, colorless solution.

### 4.4. Preparation of Eutectogel

A craft knife was used to cut two square polyethylene terephthalate (PET) films with a side length of 8 cm. They were placed separately on two square glass slides with a side length of 8 cm. Then a craft knife was used to cut a square silicone rubber sheet with a side length of 8 cm and a thickness of 1.5 mm. The silicone rubber sheet was placed between the two PET films. Then the PET films were placed sandwiching the silicone rubber sheet between the two glass slides. Six clamps were used to clamp the edges of the glass slides. Finally, a clean 5 mL syringe was used to draw the prepared solution from the glass bottle. The silicone rubber sheet was pierced with the needle and the solution injected from the syringe into the mold. Finally, the injected mold was placed into a UV light box and irradiated under a 365 nm UV light for 2 h until the solution transformed from liquid to solid.

### 4.5. Mechanical Test of Eutectogel

The mechanical strength performance of eutectogels was tested using a computer-controlled electronic universal testing machine. Each test was performed with three independent samples to reduce the impact of random errors. The testing conditions were at 25 °C, using a 100 N range sensor, performing tensile tests at a constant strain rate of 100 mm min^−1^. To prevent systematic fracture of the specimen near the fixtures, samples were cut into dumbbell shapes using a punch machine, with specific dimensions of length of 10 mm, width of 2 mm, and thickness of 1.5 mm. This method effectively ensured the integrity of the specimens and the accuracy of the test results. For fracture tensile measurements, the specimen was stretched on the fixtures at a constant strain rate of 100 mm min^−1^ until fracture, and the stress and strain at fracture were recorded. The formulas for calculating stress and strain are:

Stress: σ = F/S_0_ (3)

Strain: ε = (L − L_0_)/L_0_ × 100% (4)

where

σ: stress (MPa);

ε: strain (%);

F: tensile force applied to the gel (N);

S_0_: initial cross-sectional area of the gel (mm^2^);

L: length of the gel at fracture (mm);

L_0_: initial length of the gel (mm).

To achieve the ideal eutectogel, the optimal ratio of AA to DES solution in the gel system must first be determined.

### 4.6. Preparation of Constructing Strain Sensor

The prepared eutectogel was cut into a rectangle with a width of 0.8 cm and a length of 4 cm. Then two copper wire leads were placed at positions 1 cm from the ends on the left and right sides of the eutectogel (leaving 1 cm at each end for facilitating subsequent stretching sensing tests). In this configuration, copper wires serve as the metal electrodes for the resistive sensor. To prevent the copper wire leads from slipping during testing, which could cause excessive experimental error, the copper wire leads were fixed onto the gel with 3M tape. The other end of the copper wire leads were connected to the LCR digital bridge for testing. The as-prepared flexible sensor was secured at the testing location using 3M tape to ensure conformal attachment to the monitored subject. Real-time ΔR/R_0_ was derived from current-time data.

## Data Availability

The related data of this paper is data that should not be shared and can be obtained from the author for reasonable reasons. The author’s contact information: pjp@fosu.edu.cn.
